# Gene Alterations of N6-Methyladenosine (m^6^A) Regulators in Colorectal Cancer: A TCGA Database Study

**DOI:** 10.1155/2020/8826456

**Published:** 2020-12-19

**Authors:** Qian Zhang, Yuping Cai, Vadim Kurbatov, Sajid A. Khan, Lingeng Lu, Yawei Zhang, Caroline H. Johnson

**Affiliations:** ^1^Department of Colorectal Surgery, Second Affiliated Hospital of Harbin Medical University, Harbin, Heilongjiang Province 150086, China; ^2^Heilongjiang Academy of Medical Sciences, Harbin, Heilongjiang Province 150086, China; ^3^Department of Environmental Health Sciences, Yale School of Public Health, 60 College Street, New Haven, CT 06520-8034, USA; ^4^Department of Surgery, Yale School of Medicine, New Haven, CT 06510, USA; ^5^Section of Surgical Oncology, Department of Surgery, Yale School of Medicine, New Haven CT 06510, USA; ^6^Department of Chronic Disease Epidemiology, Yale School of Public Health, Yale University, 60 College Street, New Haven, CT 06520-8034, USA

## Abstract

N6-methyladenosine (m^6^A) plays an important role in many cancers. However, few studies have examined the role of m6A in colorectal CRC. To examine the effect of m6A on CRC, we studied the genome of 591 CRC cases from The Cancer Genome Atlas (TCGA). The relationship between the messenger RNA (mRNA) expression, copy number variation (CNVs), and mutations of m6A “Writers,” “Readers,” and “Erasers,” prognosis, immune cell infiltration, and genetic mutations in CRC cases were analyzed. CNVs and mutations were found in thirteen m6A regulators. As expected, gain and amplification of m6A regulators increased the mRNA expression of these regulators, while deletion led to reduction in the mRNA expression. Moreover, CNVs and mutation of these regulators were significantly associated with APC, TP53, and microsatellite instability (MSI) status (*p* < 0.001, *p* < 0.001, and *p* = 0.029, respectively). CNVs of m6A regulators also correlated with inferred immune cell infiltration in CRC tissues, especially in colon tissues. Additionally, alterations of RBM15, YTHDF2, YTHDC1, YTHDC2, and METTL14 genes were related to the worse overall survival and disease-free survival (DFS) of CRC patients. Specifically, the deletion status of “Writers” was also correlated to the DFS of CRC patients (*p* = 0.02). Gene set enrichment analysis found that FTO was involved in mRNA 3′ end processing, polyubiquitin binding, and RNA polymerase promoter elongation, while YTHDC1 was related to interferon-alpha and gamma response. In conclusion, a novel relationship was identified between CNVs and mutations of m6A regulators with prognosis and inferred immune function of CRC. These findings will improve the understanding of the relationship of m6A in CRC.

## 1. Introduction

Colorectal cancer (CRC) is one of the most lethal malignant diseases worldwide. It is the third leading cause of cancer-related death and third most commonly diagnosed cancer in both men and women in the United States [1]. Despite many studies clarifying the tumor biology of CRC, the incidence and mortality of patients with CRC are still high. Therefore, effective solutions are urgently needed for earlier diagnosis and to predict prognosis.

The concept of epigenetics was first discussed in 1942; since then, epigenetics has been proven to play vital roles in carcinogenesis and cancer progression [2]. RNA modifications are considered as a kind of epigenetics [3] and currently, more than 170 RNA modifications are recognized [4]. Cellular RNAs (rRNAs, transfer RNAs (tRNAs), mRNAs, small nuclear RNAs (snRNAs), lncRNAs and miRNAs, and others) contain over a hundred structurally distinct posttranscriptional modifications at thousands of sites [3]. It has been discovered that RNA modifications regulate most steps of the gene expression, from DNA transcription to mRNA translation [5, 6]. It was not until recently that expansive enthusiasm for RNA modification resurged, provoked by identification of internal mRNA modification, most conspicuously N6-methyladenosine (m6A), methylated at the N6 position of adenosine.

M6A has been the most studied RNA modification to date. It was first reported as the main pattern of eukaryotic mRNA methylation [7]. M6A modification on RNA is abundant near the stop codon and 3′-untranslated region (3′-UTR) [8, 9] and translated near 5′-UTR in a cap-independent manner [10], thereby regulating RNA transcription, translation, and metabolism. The effectors in m6A pathways include “writers” and “erasers” that install and remove the methylation and “readers” that recognize it, respectively. “Writers” include methyltransferase-like 3 (METTL3) [11], METTL14 [12], Wilms tumor 1-associated protein (WTAP) [13], RBM15/15B [14], and KIAA1429 (VIRMA) [15], which introduce the methyl code to target RNAs; “Erasers” mainly include fat mass and obesity-associated protein (FTO) [16] and alkB homologue 5 (ALKBH5) [17], which both selectively delete the methyl code from target RNAs; ‘Readers' such as YT521-B homology (YTH) domain containing 1 (YTHDC1), YTHDC2 [18], YTH N6-methyl-adenosine RNA binding protein 1 (YTHDF1), YTHDF2 [19], eukaryotic initiation factor (eIF) 3 [14], IGF2 mRNA binding proteins (IGF2BP) families [20], heterogeneous nuclear ribonucleoprotein (HNRNP) protein families [21], and zinc finger CCCH domain-containing protein 13 (ZC3H13) [22] can decipher the m6A methylation code.

Growing evidence suggests that m6A modification has been playing an important role in various cancers. This modification is closely linked to increased tumor proliferation, carcinogenesis, migration, and metastasis [23, 24]. In CRC, it has been reported that METTL3 could facilitate tumor progression [25], and YTHDF1 regulates tumorigenicity and cancer stem cell-like activity [26]. Also, m6A modification is related to the lncRNA RP11 activity to trigger the dissemination of CRC cells via upregulation of Zeb1 [27]. RNA modification genes have also been shown to modify T cell activation in cancers. However, there are relatively few studies on the extensive role of m6A RNA modification regulators in CRC prognosis, immune status, and other clinicopathological features. We hypothesize that m6A modification is widely involved in CRC.

This study is aimed at improving the understanding of m6A in CRC and providing some evidence for future examination of the role of RNA m6A methylation in CRC.

## 2. Materials and Methods

### 2.1. Datasets

The clinical information, copy number variation (CNV) data, somatic mutation data, and RNA expression data were obtained from The Cancer Genome Atlas (TCGA) cBioportal platform (https://www.cbioportal.org/). The data about immune cell infiltration in tissues was obtained from TIMER (https://cistrome.shinyapps.io/timer/) [28].

### 2.2. Data Grouping and Analysis

CRC cases with CNV, mutation, and clinicopathological information were retrieved from the TCGA database (Colorectal Adenocarcinoma Project). CNV was identified using segmentation analysis and GISTIC algorithm in the cBioportal platform. The relationship between clinicopathological characteristics was analyzed according to the status of CNV and/or mutation: “patients with mutation and/or CNV of m6A regulators” and “patients without CNV or mutation.” For the RNA-seq data, mRNA expression *Z*-scores, RSEM (Batch normalized from Illumina HiSeq_RNASeqV2), were obtained from the cBioportal platform. Then, the mRNA expression level was analyzed according to the CNV status of m6A genes. Gene set enrichment analysis (GSEA) was performed using GSEA 3.0 (http://software.broadinstitute.org/gsea/index.jsp) in which the hallmark gene set “h.all.v6.0.symbols.gmt” was adopted. In this study, cases were divided into two groups according to the median expression of m6A regulators' mRNA. Gene sets with a nominal *p* value <0.05 and the false discovery rate (FDR) < 0.25 were considered to be significantly enriched.

### 2.3. M^6^A Regulatory Gene Selection

A comprehensive method was adopted to identify m6A regulatory genes. First, a list of m6A regulators was retrieved from published literature, and then the list was mapped into the TCGA database to exclude the genes of which the data are not available in the database. In total, thirteen m6A RNA modification genes were identified. “Writer”: METTL3, METTL14, WTAP, VIRMA, and RBM15; “Eraser”: FTO and ALKBH5; “Reader”: ZC3H13, YTHDC1, YTHDC2, YTHDF1, YTHDF2, and HNRNPC.

### 2.4. Statistical Analysis

Data and figures were analyzed using *R* programming version 3.6.1. The chi-square test or Mann–Whitney *U* test was used to analyze the correlation between m6A regulators and clinicopathological characteristics. The Kaplan-Meier curve and log-rank test were used to detect the effect of m6A regulatory genes on prognosis. Cox proportional hazard regression models were performed. The overall survival (OS) and disease-free survival (DFS) were defined as the number of months from the initial diagnosis until death or recurrence or the last follow-up, respectively. The infiltration level of immune cells in cancer tissues for each CNV category was compared with those in the normal tissues using a two-sided Wilcoxon rank-sum test in the immune cells' infiltration analysis.

## 3. Results

### 3.1. M^6^A Regulatory Gene CNV Status

**Figure 1:**
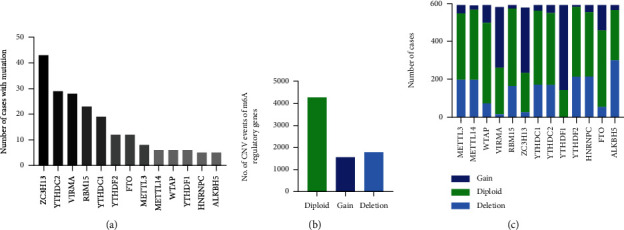
CNVs and mutation events of m6A regulators. (a) Mutation events of m6A regulators in the cohort. (b) Total CNV events of m6A regulators in the cohort. (c) CNV distribution of each m6A regulator.

591 cases with CNV and mutation data were included in our study among which CNVs were observed in all of the m6A regulator genes. Mutations were observed in all of the thirteen m6A regulators, however, only in a small number of the cases (Figure 1(a)). Moreover, the number of deletion CNV events (1797) was almost equal to the number of gain events (1573) (Figure 1(b)). Notably, 76.1% of the cases harbored YTHDF1 CNVs which was the most commonly observed among all the m6A regulators, while only 28.26% of cases acquired WTAP CNVs (Figure 1(c)).

### 3.2. CNVs of m^6^A Regulators with Clinicopathological and Molecular Features

To fully explore the significance of CNVs/mutation of m6A regulators in CRC, we next questioned whether CNVs are associated with clinicopathological characteristics. Age, sex, tumor stage, vascular invasion, lymphovascular invasion, perineural invasion, and sidedness were evaluated. The data revealed that tumor staging was related with CNVs/mutation of m6A regulatory genes, and there was a borderline significance (*p* = 0.058) between M staging and CNV/mutation status (Table 1). To further understand the mechanisms underlying CNVs/mutation of the m6A regulatory genes, we analyzed the relationship between CNVs/mutation and common gene mutation in CRC, such as KRAS, NRAS, APC (Adenomatous polyposis coli), and TP53. The data showed that APC and TP53 mutations were significantly associated with m6A regulator CNVs/mutation which is consistent with previous data [29], indicating that cases with APC or TP53 mutation were prone to have the m6A regulatory gene CNVs/mutation (Table 2). We also analyzed the relationship between the MSI status and CNVs/mutation, and data showed that MSS cases were prone to have the m6A modification genes' CNVs/mutation (Table 2).

**Figure 2:**
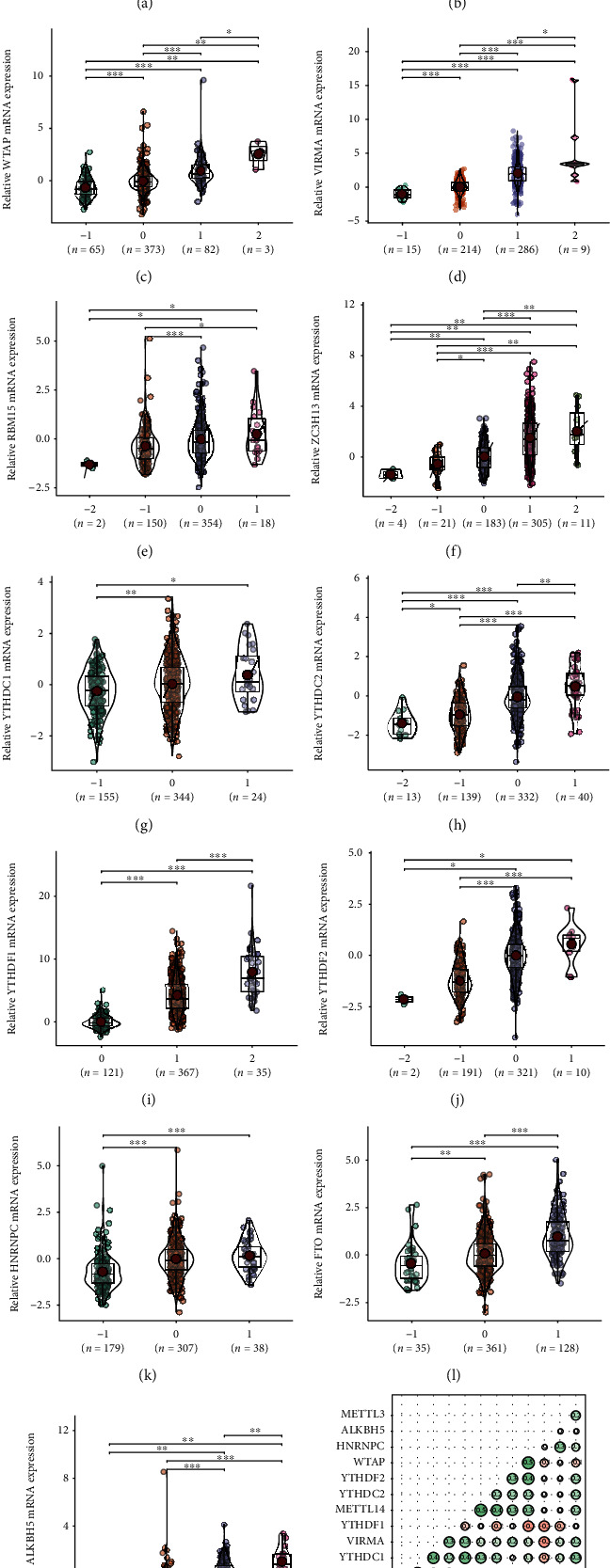
(a)–(m) Effect of CNVs on the mRNA expression of each m6A regulator (-2: deep deletion; -1: shallow deletion; 0: diploid; 1: gain; 2: amplification). (n) mRNA correlation among thirteen m6A regulators. ^∗^*p* < 0.05; ^∗∗^*p* < 0.01; ^∗∗∗^*p* < 0.001.

To investigate whether CNVs/mutations affected the mRNA expression of m6A regulators, we then examined the relationship between the CNV status and individual m6A regulator mRNA expression in cases with the mRNA expression data. As expected, copy number loss of m6A regulators was associated with lower expression of mRNAs, while copy number gains were related with the higher expression of these mRNAs (Figures 2(a)–2(m)). We also analyzed the correlation of the mRNA expression among all of the thirteen m6A regulators. It suggested that YTHDC1 was correlated with several other genes including VIRMA, METTL14, ZC3H13, and FTO, indicating a central role of YTHDC1 in the m6A modification. In addition, three pairs of genes, WTAP/HNRNPC, METTL14/YTHDC2, and FTO/ZC3H13, were also closely correlated with each other (Figure 2(n)).

### 3.3. Immune Cell Infiltration and CNVs of m6A Regulators

**Figure 3:**
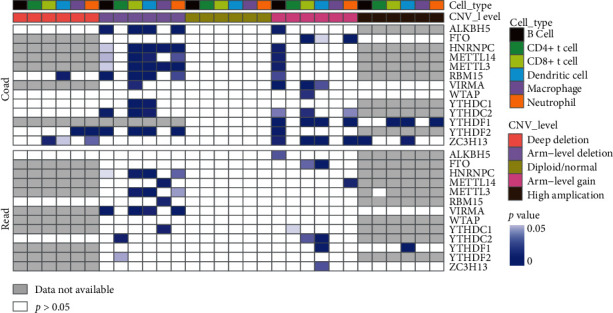
CNVs affect immune cell infiltration in CRC tissues. Blue grid represents that CNV is statistically involved in immune cell infiltration in cancer tissues compared with normal tissues. Grey grid represents that data is not available while white grid represents that *p* > 0.05. COAD: colon adenocarcinoma; READ: rectum adenocarcinoma.

The immune system plays an important role in carcinogenesis, especially in therapeutic efficacy of immunotherapy. The tumor microenvironment (TME) often contains large numbers of infiltrating myeloid cells including monocytes, macrophages, dendritic cells, and granulocytes. These cells exert various functions in the TME, ranging from regulating the immune process to drug sensitivity. Therefore, we examined whether CNVs of m6A regulators were linked with changes to inferred immune cells infiltration. As shown in Figure 3, CNVs of m6A regulators were closely correlated with immune cell infiltration in the COAD (colon adenocarcinoma) cohort, while in the READ (rectal adenocarcinoma) cohort, the effect of CNVs of m6A genes was reduced. The published literature on the m6A modification also reveals that the m6A modification is related to immune cell activation [30], homeostasis [28], and immune response [31].

### 3.4. CNVs of m6A Regulators with the Prognosis of CRC Patients

**Figure 4:**
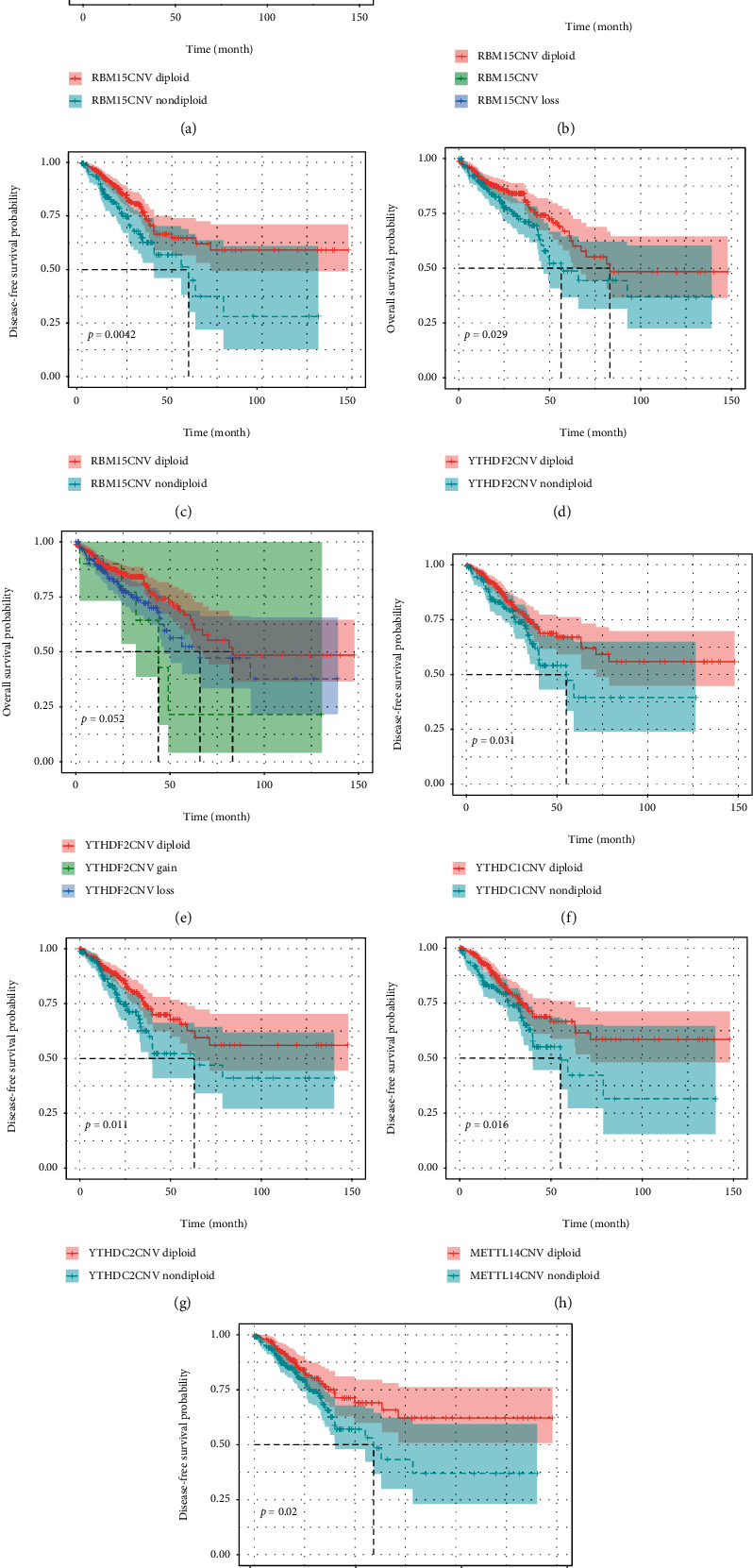
Relationship between CNVs of m6A regulators and OS and DFS. OS: overall survival; DFS: disease-free survival.

We next addressed the relationship between prognosis and m6A regulatory genes to better interpret the role of m6A regulators. The data indicated that RBM15 played an important role in the CRC outcome. Cases with nondiploid (loss/gain) CNVs of RBM15 had a worse OS (HR = 1.89, *p* = 0.001) and DFS (HR = 1.78, *p* = 0.005) rates compared with patients who had diploid CNV in the three genes (Figures 4(a) and 4(c)). Moreover, when patients were divided into three groups according to loss, gain, and diploid CNV status, it showed that patients with gain of CNVs in RBM15 had the worse OS compared with patients that had loss or diploid CNV status (gain: HR = 2.57, *p* = 0.018; loss: HR = 1.91, *p* = 0.003) (Figure 4(b)). In addition, nondiploid YTHDF2 yielded poorer OS compared to diploid YTHDF2 (HR = 1.43, *p* = 0.064) (Figure 4(d)). However, there was only a borderline significance for patients that had gain of YTHDF2 mRNA that led to a worse OS when compared with loss and diploid CNVs of YTHDF2 (Figure 4(e)). Moreover, alterations of YTHDC1, YTHDC2, and METTL14 were linked to poorer DFS as shown in Figures 4(f)–4(h). As “Writer” loss was related with poor DFS as shown in Table 3, we further stratified the patients according to the CNV status of “Writer.” The data suggested that patients with gain of “Writer” had better DFS than those without indicating that the upregulation of “Writer” is related to good survival outcome of CRC (Figure 4(i)).

To test whether CNVs of m6A regulatory genes were independent prognostic factors, cox regression analysis was performed. The results suggested that “Writer” loss positive/“Eraser” gain positive was associated with poorer DFS; however, it was not an independent factor according to the multivariate analysis. In addition, age, tumor stage, tumor stage, T/N/M stage, MSS (microsatellite stable), vascular invasion, lymph-vascular invasion, and lymph node count were associated with OS. In addition, age, tumor stage, and lymph node count were independent factors of OS indicated by multivariate analysis. Tumor stage, T/N/M stage, vascular invasion, lymph-vascular invasion, and “Writer” loss status/“Eraser” gain status were correlated with DFS, and only the lymph-node count was an independent factor after multivariate analysis (Table 3).

### 3.5. Gene Set Enrichment Assay (GSEA)

**Figure 5:**
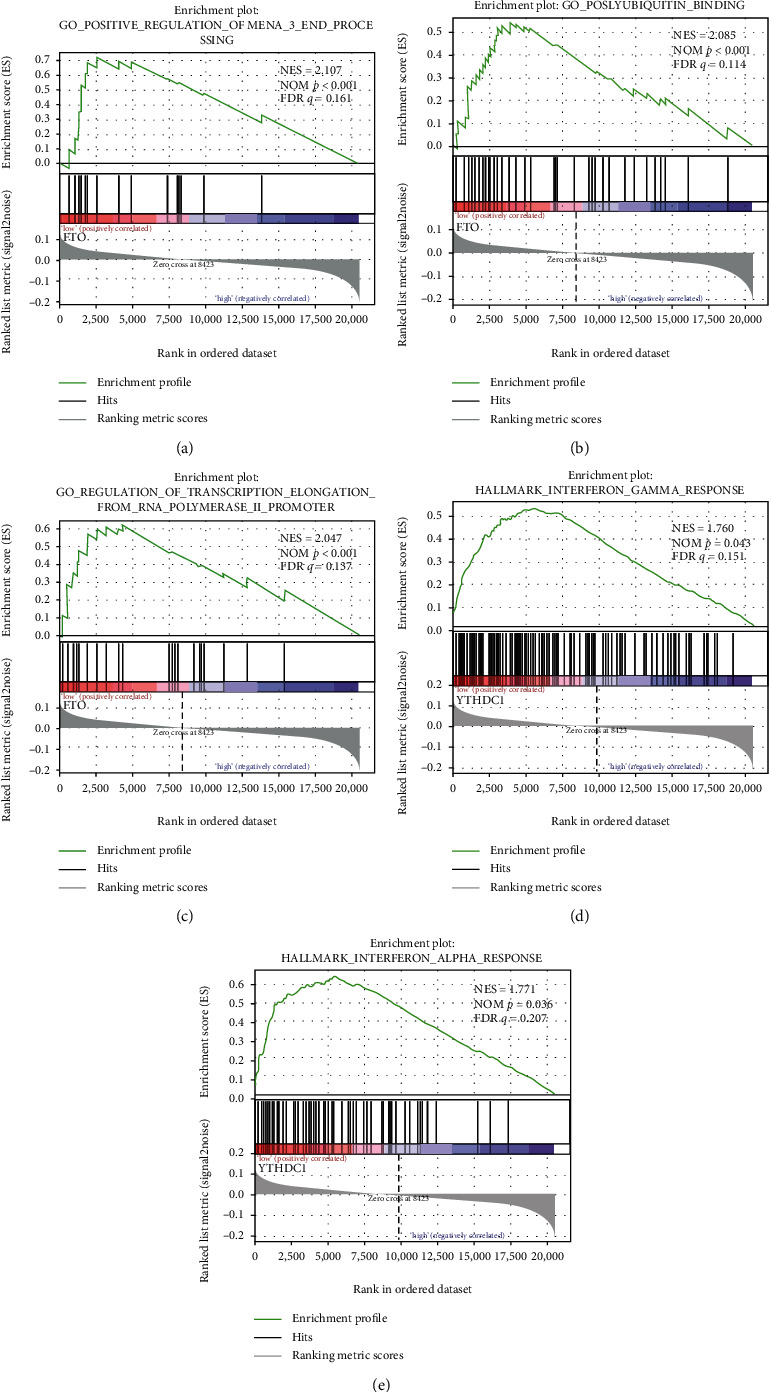
GSEA results of the mRNA level of FTO and YTHDC1. Gene set enrichment plots of (a) mRNA 3′ end processing, (b) polyubiquitin binding, (c) transcription from RNA polymerase promoter, (d) interferon-gamma, and (e) interferon-alpha response.

Due to the importance of CNVs in m6A regulators in carcinogenesis, we next questioned whether specific pathways are changed by these m6A regulatory genes. We explored the enriched gene sets in samples with low or high m6A regulatory gene mRNA expression levels in 524 cases. The GSEA results implied that the FTO low expression was associated with polyubiquitin binding, mRNA 3′ end processing, and transcription elongation from RNA polymerase II (Figures 5(a)–5(c)). These biological processes are widely involved in tumors' malignancy [32–34]. In addition, the YTHDC1 low expression was related with the interferon-gamma response and interferon-alpha (Figures 5(d) and 5(e)).

## 4. Discussion

Previous TCGA studies have assessed genomic correlation [35], immune cells infiltration, and molecular characterization [36] of CRC. In our study, we comprehensively explored the effect of CNVs and mutations in m6A regulators on the mRNA expression, immune cell infiltration, metabolic pathways, and CRC patient survival. We observed that CNVs affected the mRNA expression of m6A regulators, immune cell infiltration in CRC tissues, and patient prognosis. Furthermore, the dysregulated mRNA expression correlated with immune cell regulatory pathways.

Our data shows that the majority of CRC patients had acquired CNV or mutations to m6A modification genes, with 76%, 64%, and 58% cases harboring YTHDF1, ZC3H13, and VIRMA CNV, respectively. The frequency of alterations is much higher than other cancers, such as acute myelocytic leukemia [29] and renal cancer [37]. It suggests that the m6A modification plays a more important role in CRC. It has been shown that various m6A correlated genes are involved in the regulation of carcinogenesis, proliferation, migration, and stem cell-like activity [25–27]. Interestingly, “Reader” ZC3H13 and “Writer” VIRMA are both predisposed to mutation and copy number gain, as positively correlated with each other as shown in Figure 2(n).

An important discovery was the significant association between APC and TP53 mutations and CNV and mutations to m6A modification genes. APC and TP53 mutations are common in CRC. APC is a tumor suppressor gene frequently mutated in CRC. Mutation and inactivation of this gene are a key, and early event almost uniquely observed in colorectal tumorigenesis. The correlation between m6A regulators and APC mutation suggests the role of the m6A modification in the early stage of CRC. Consistently, in AML patients, it has been reported that mutations and CNVs of m6A regulators are related to TP53 mutations [29]. As epigenetic regulations unlikely lead to genomic alterations, it is reasonable that alterations of m6A genes are induced by other functional events, probably through upregulation of cancer promoters or downregulation of tumor inhibitors. Interestingly, the MSI status was also correlated to CNV and mutations in m6A regulators.

Different consensus molecular subtype (CMS) classifications have been developed to facilitate clinical translation [38], among which CMS1 (MSI Immune) is featured with hypermutated, microsatellite instable, and strong immune activation [38]. MSI is a crucial biomarker in the prognosis of CRC, and MSI-high (MSI-H) status is associated with a better prognosis compared to MSS CRC [39]. The analysis of the MSI status and m6A genes' CNVs and mutation showed that cases with MSS are prone to CNVs and mutations to m6A regulators. Additionally, cases with nondiploid m6A genes have a poorer prognosis as shown in Figure 4, which is consistent with that patients harboring MSS that have a worse survival compared with those with MSI-H [40]. These data warrant further study about the biological and clinical roles of m6A genes alteration in CRC.

Right-sided and left-sided colon cancer exhibit different molecular features. Left-sided colon cancer (LCC) harbors more chromosomal instability pathway-related mutations, including APC, KRAS, and P53 mutations, while MSI and DNA mismatch repair pathways are commonly observed in the right-sided tumors (RCC) [41]. We found m6A regulator CNVs/mutation is statically related with MSS, APC mutation, and p53 mutation which are features of left-sided cancer. However, there is no significant correlation between the regulators CNVs/mutation and sidedness of colon cancer. This could be due to the limited sample size in our study or too much missing information regarding the MSI status.

CRC is dominated by diverse and plastic immune cell infiltration. These immune cells have an important effect on tumor development. Some populations of cells are strongly correlated with DFS and OS [38]. Also, genomic correlates of immune cell infiltrate in colorectal carcinoma [35]. In our study, we analyzed immune cell infiltration and the CNV status of m6A regulators. Nondiploid CNV status (deletion and gain) has a significant effect on CRC immune cell infiltration. B cells, CD8+ T cells, macrophages, neutrophils, and dendritic cells are affected by gene deletion or gain. It is consistent with previous studies that show macrophages, dendritic cells, neutrophils, B cells, and T cells which are involved in the outcomes of CRC [42]. It is reported that METTL3 could facilitate macrophage polarization through the methylation of STAT1 mRNA [43], and mettl3-mediated m6A methylation promotes dendritic cell activation [30]. Moreover, YTHDF1 has been proved to be related with the antigen-specific CD8+ T cell antitumor response [44]. It is not surprising that patients from the COAD cohort have higher susceptibility to CNVs than those in the READ when it comes to immune cell infiltration. Colon and rectal cancers differ from each other in terms of anatomic location of the tumor, prognosis, recurrence rate, and treatment strategy. Additionally, the immune profile of colon is different from that of rectum. It is reported that CD3+ T lymphocytes, CD8+ T lymphocytes, and the effector molecule granzyme B infiltration are correlated with OS of colon cancer but not rectal cancer [45], while lymphocytic infiltration is associated with relapse and distant metastasis of rectal cancer but not colon cancer according to the study of Nagtegaal et al. [46]. These data illustrate the role of immune systems in CRC and CNVs of m6A regulators that could be used as a promising target to treat CRC.

In conclusion, we systematically demonstrated the effect of CNVs and mutations to m6A regulators on the mRNA expression, immune cell infiltration, prognosis, and metabolic pathway switch of CRC. Our study provides evidence for future study on the role of RNA m6A methylation in CRC and therapeutic target to treat CRC.

## Figures and Tables

**Table 1:** CNVs/Mutation of m6A regulators with clinicopathological characteristics.

	Without mutation/CNV	With mutation/CNV	*p* value
Age			0.583
≤ 65	16	284	
> 65	11	263	
Gender			0.761
Male	12	300	
Female	13	264	
Stage			0.045
I	4	98	
II	15	202	
III	6	167	
IV	0	82	
T stage			0.938
Tis	0	1	
T1	0	19	
T2	5	98	
T3	17	384	
T4	3	62	
N stage			0.222
N0	19	319	
N1	3	141	
N2	3	103	
M stage			0.058
M0	21	421	
M1	0	79	
Primary site^∗^			0.307
RCC	10	175	
LCC	5	177	
Vascular invasion			1
Without	13	378	
With	4	118	
Lymphovascular invasion			0.821
Without	13	306	
With	7	205	
Perineural invasion			0.680
Without	6	157	
With	1	56	

^∗^RCC: right-sided colon cancer; LCC: left-sided colon cancer.

**Table 2:** Correlation of m*^6^*A regulatory genes' CNVs/Mut and common genes' mutation.

		Without MUT/CNV	With MUT/CNV	*p*
APC	WT	14	126	<0.001
	MUT	11	440	
TP53	WT	21	203	<0.001
	MUT	4	363	
KRAS	WT	17	321	0.363
	MUT	8	245	
NRAS	WT	21	530	0.141
	MUT	4	36	
MSI status	MSI	7	68	0.029
	MSS	4	174	

WT: wild type; MUT: mutation; MSI: microsatellite instability; MSS: microsatellite stable; CNV: copy number variation.

**Table 3:** Univariate and multivariate COX regression analysis of m*^6^*A regulatory genes for CRC patients' OS and DFS.

Characteristics	OS				DFS			
	Univariate		Multivariate		Univariate		Multivariate	
	HR (95% CI)	*p*	HR (95% CI)	*p*	HR (95% CI)	*p*	HR (95% CI)	*p*
Age (>65 vs ≤65)	2.5 (1.68-3.72)	0.000	2.61 (1.08-6.28)	0.033	1.05 (0.72-1.53)	0.811	1.09 (0.70-1.71)	0.700
Sex (male vs female)	1.07 (0.75-1.53)	0.707			1.35 (0.91-1.98)	0.132		
Tumor stage								
I	1				1		1	
II	1.62 (0.71-3.68)	0.249	NA (0-inf)	0.998	2.22 (0.99-4.99)	0.054	2.16 (0.38-12.47)	0.388
III	3.07 (1.37-6.88)	0.006	NA (0-inf)	0.997	3.62 (1.61-8.12)	0.002	3.17 (0.45-22.27)	0.246
IV	7.96 (3.55-17.86)	0.000	NA (0-inf)	0.997	8.45 (3.66-19.48)	<0.001	1.08 (0.07-16.48)	0.959
T stage								
T1	1				1		1	
T2	0.83 (0.18-3.92)	0.816	NA (0-inf)	0.999	0.92 (0.2-4.25)	0.914	1.16 (0.14-9.88)	0.890
T3	1.92 (0.47-7.8)	0.363	NA (0-inf)	0.999	2.05 (0.5-8.36)	0.315	1.03 (0.09-11.36)	0.980
T4	5.92 (1.4-25.06)	0.016	NA (0-inf)	0.999	6.19 (1.45-26.54)	0.014	3.01 (0.27-34.02)	0.370
N stage								
N0			1		1		1	
N1	1.77 (1.13-2.78)	0.013	0.11 (0.02-0.63)	0.013	1.8 (1.14-2.84)	0.011	0.86 (0.26-2.86)	0.805
N2	4 (2.63-6.08)	<0.001	0.45 (0.09-2.32)	0.338	3.58 (2.27-5.64)	<0.001	1.81 (0.54-6.05)	0.334
M stage (M1 vs M0)	4.28 (2.85-6.42)	<0.001			3.59 (2.26-5.68)	<0.001	6.97 (0.78-62.58)	0.083
Site								
Abdomen	1				1			
Colon	0.42 (0.06-3.02)	0.389			0.35 (0.05-2.54)	0.301		
Rectum	0.32 (0.04-2.42)	0.267			0.36 (0.05-2.72)	0.323		
TP53 (wild type vs mutant)	0.97 (0.67-1.4)	0.860			0.91 (0.61-1.35)	0.630		
APC (wild type vs mutant)	1.08 (0.7-1.66)	0.719			1.27 (0.82-1.97)	0.276		
KRAS (wild type vs mutant)	1.17 (0.81-1.67)	0.408			0.7 (0.48-1.03)	0.070	0.755 (0.48-1.19)	0.223
NRAS (wild type vs mutant)	0.86 (0.44-1.7)	0.663			1.55 (0.63-3.81)	0.339		
BRAF (wild type vs mutant)	0.76 (0.46-1.23)	0.264			1.12 (0.6-2.08)	0.729		
MSI status (MSS vs MSI)	0.52 (0.29-0.93)	0.027	0.31 (0.13-0.76)	0.01	0.79 (0.39-1.62)	0.526		
Vascular invasion (with vs without)	2.28 (1.52-3.41)	0.000	0.74 (0.31-1.77)	0.494	1.77 (1.14-2.75)	0.012	1.18 (0.64-2.17)	0.598
Lymphovascular invasion (with vs without)	2 (1.36-2.95)	0.000	1.86 (0.68-5.11)	0.228	1.66 (1.12-2.47)	0.012	0.78 (0.42-1.44)	0.420
Perineural invasion (with vs without)	1.56 (0.8 - 3.03)	0.188			1.61 (0.83-3.12)	0.157		
Lymph node count								
0-11	1		1		1		1	
12-23	0.55 (0.34-0.89)	0.016	0.67 (0.10-4.70)	0.688	0.68 (0.38-1.2)	0.181	2.32 (1.13-4.74)	0.020
> 24	0.46 (0.27-0.77)	0.003	0.18 (0.02-1.51)	0.114	0.58 (0.32-1.06)	0.076	1.44 (0.88-2.36)	0.140
CNV/MUT (with vs without)	0.66 (0.31-1.43)	0.295			1.02 (0.38-2.78)	0.964		
WTAPCNV								
Deep deletion	1		1					
Shallow deletion	0.13 (0.02-1)	0.050	1.92 (0.15-24.62)	0.615	1			
Diploid	0.08 (0.01-0.62)	0.015	0.43 (0.03-5.85)	0.528	0.7 (0.41-1.2)	0.189		
Gain	0.11 (0.01-0.82)	0.032	0.47 (0.04-6.24)	0.57	0.9 (0.45-1.78)	0.764		
Amplification	0.19 (0.01-3.11)	0.247	NA (0-inf)	0.999	1.43 (0.19-10.83)	0.727		
Writer loss eraser gain status								
LNGN	1		1		1		1	
LNGP	0.64 (0.29-1.42)	0.273	0.51 (0.10-2.48)	0.4	1.04 (0.48-2.27)	0.917	1.19 (0.46-3.07)	0.730
LPGN	1.43 (0.95-2.13)	0.083	0.59 (0.21-1.67)	0.316	1.54 (0.97-2.43)	0.066	1.58 (0.90-2.76)	0.110
LPGP	0.87 (0.48-1.57)	0.637	0.22 (0.04-1.09)	0.064	1.77 (1.03-3.03)	0.038	1.64 (0.85-3.14)	0.140

CNV: copy number variation; LN(P)GN(P): loss negative (positive) gain negative (positive). OS: overall survival; DFS: disease-free survival.

## Data Availability

The data used to support the findings of this study are available in the Cancer Genome Atlas database (https://www.cancer.gov/about-nci/organization/ccg/research/structural-genomics/tcga).
